# Dog10K: an international sequencing effort to advance studies of canine domestication, phenotypes and health

**DOI:** 10.1093/nsr/nwz049

**Published:** 2019-04-10

**Authors:** Elaine A Ostrander, Guo-Dong Wang, Greger Larson, Bridgett M vonHoldt, Brian W Davis, Vidhya Jagannathan, Christophe Hitte, Robert K Wayne, Ya-Ping Zhang, Dog10K Consortium, Catherine André, Erik Axelsson, Adam Boyko, Brian W Davis, Oliver Forman, Laurent Frantz, Christophe Hitte, Vidhya Jagannathan, Elinor Karlsson, Jeffrey Kidd, Greger Larson, Tosso Leeb, Kerstin Lindblad-Toh, Hannes Lohi, Kirk E Lohmueller, Tomas Marques-Bonet, Catherine Mellersh, *Elaine A Ostrander, Peter Savolainen, Robert Schnabel, Bridgett M vonHoldt, *Guo-Dong Wang, *Robert K Wayne, Ziheng Yang, Weiwei Zhai, *Ya-Ping Zhang

**Affiliations:** 1 National Human Genome Research Institute, National Institutes of Health, Bethesda, MD 20892, USA; 2 State Key Laboratory of Genetic Resources and Evolution, Kunming Institute of Zoology, Chinese Academy of Sciences, Kunming 650223, China; 3 Center for Excellence in Animal Evolution and Genetics, Chinese Academy of Sciences, Kunming 650223, China; 4 Palaeogenomics and Bio-Archaeology Research Network, School of Archaeology, University of Oxford, Oxford OX1 3TG, UK; 5 Department of Ecology and Evolutionary Biology, Princeton University, Princeton, NJ 08544-1014, USA; 6 College of Veterinary Medicine and Biomedical Sciences, Texas A&M University, College Station, TX 77840, USA; 7 Institute of Genetics, Vetsuisse Faculty, University of Bern, Bern CH-3001, Switzerland; 8 IGDR, CNRS, University of Rennes, Rennes F-3500, France; 9 Department of Ecology and Evolutionary Biology, University of California, Los Angeles, Los Angeles, CA 90095, USA; 10 University of Rennes, CNRS, IGDR, UMR6290 - F-3500 Rennes, France; 11 Department of Medical Biochemistry and Microbiology, Uppsala University, Uppsala biomedicinska centrum BMC, Husarg. 3 75237 Uppsala, Sweden; 12 Department of Biomedical Sciences, Cornell University College of Veterinary Medicine, Veterinary Research Tower T8016A, Box 17, Ithaca, NY 14853, USA; 13 College of Veterinary Medicine and Biomedical Sciences, Texas A&M University, College Station, TX, USA; 14 Genetics Research Unit, Mars Inc., Cambridge, UK; 15 School of Biological and Chemical Sciences, Queen Mary University of London, London, UK; 16 University of Rennes, CNRS, IGDR - UMR6290, F-3500 Rennes, France; 17 Institute of Genetics, Vetsuisse Faculty, University of Bern, Bern, Switzerland; 18 Bioinformatics and Integrative Biology at University of Massachusetts Medical School, and Vertebrate Genomics and Genetics Group at the Broad Institute of the Massachusetts Institute of Technology and Harvard, Boston, MA, USA; 19 Departments of Human Genetics and Computational Medicine and Bioinformatics, University of Michigan Medical School, 3726A Med Sci II, Ann Arbor, MI 48109, USA; 20 Research Laboratory for Archaeology and the History of Art, Dysons Perrins Building, South Parks Road, University of Oxford, Oxford, OX1 3QY, UK; 22 Science for Life Laboratory, Department of Medical Biochemistry and Microbiology, Uppsala University, Uppsala, Sweden and Broad Institute of the Massachusetts Institute of Technology and Harvard, Cambridge, MA 02142, USA; 23 Research Programs Unit, Molecular Neurology and Department of Veterinary Biosciences, University of Helsinki and Folkhälsan Institute of Genetics, PO Box 63, Helsinki, Finland; 24 Ecology and Evolutionary Biology, University of California, Los Angeles, Los Angeles, CA 90095, USA; 25 Institut Biologia Evolutiva (Universitat Pompeu Fabra/CSIC) ICREA, Barcelona, Spain; 26 Animal Health Trust, Lanwades Park, Kentford, Newmarket, Suffolk, CB8 7UU, UK; 27 Room 5351, Building 50, National Human Genome Research Institute, National Institutes of Health, 50 South Drive, Bethesda, MD 20892, USA; 28 School of Biotechnology, Science for Life Laboratory, Royal Institute of Technology in Stockholm, PO Box 1031, SE-171 21, Solna, Sweden; 29 Division of Animal Sciences, Informatics Institute, University of Missouri-Columbia, Columbia, MO 65211, USA; 30 Ecology and Evolutionary Biology, Princeton University, Princeton, NJ 08544, USA; 33 Department of Genetics, University College London, London, WC1E 6BT, UK; 34 Key Laboratory of Zoological Systematics and Evolution, Institute of Zoology, Chinese Academy of Sciences, Beijing 100101, P.R. China

**Keywords:** genomics, genome-wide association studies (GWAS), breed, selection, variation, evolution

## Abstract

Dogs are the most phenotypically diverse mammalian species, and they possess more known heritable disorders than any other non-human mammal. Efforts to catalog and characterize genetic variation across well-chosen populations of canines are necessary to advance our understanding of their evolutionary history and genetic architecture. To date, no organized effort has been undertaken to sequence the world's canid populations. The Dog10K Consortium (http://www.dog10kgenomes.org) is an international collaboration of researchers from across the globe who will generate 20× whole genomes from 10 000 canids in 5 years. This effort will capture the genetic diversity that underlies the phenotypic and geographical variability of modern canids worldwide. Breeds, village dogs, niche populations and extended pedigrees are currently being sequenced, and *de novo* assemblies of multiple canids are being constructed. This unprecedented dataset will address the genetic underpinnings of domestication, breed formation, aging, behavior and morphological variation. More generally, this effort will advance our understanding of human and canine health.

## INTRODUCTION

**Figure 1.**
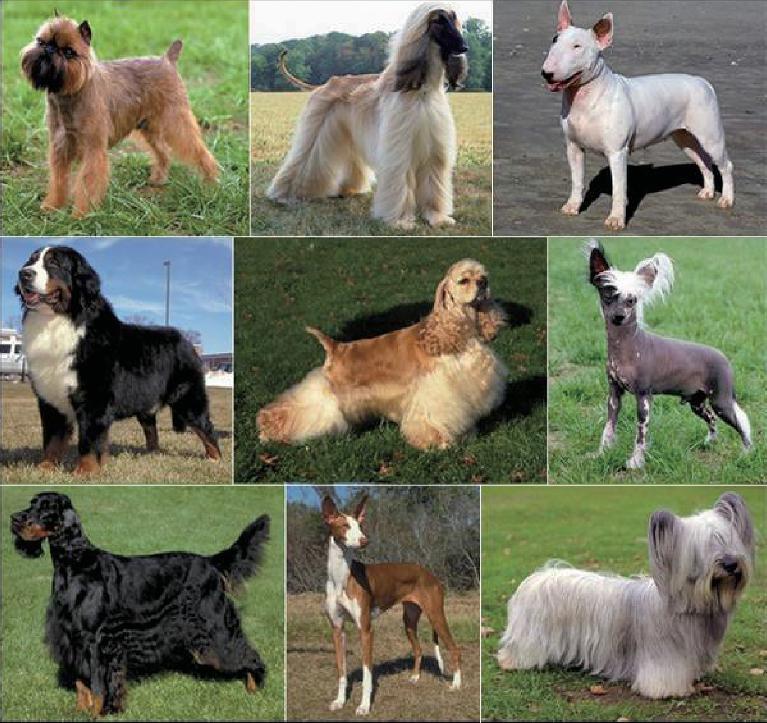
Morphological variation among established breeds. Dog breeds show extraordinary amounts of variation in size, coat color, skull shape, etc. Within a breed there are high levels of uniformity, but between breeds variation is common. Beginning at upper left and going clockwise are pictures of the following breeds: Brussels Griffon, Afghan Hound, Bull Terrier, Chinese Crested Dog, Skye Terrier, Basenji, Gordon Setter and Bernese Mountain dog, and in the center is a Cocker Spaniel.

Domestic dogs (*Canis lupus familiaris*) are the most variable mammalian species on Earth [1–3] (Fig. 1). Strong artificial selection has produced approximately 450 globally recognized breeds with distinct traits related to morphology [4] including, but not limited to, body size [4,5], tail phenotype [6], fur type [7,8], skull shape [6,9–11] and pigmentation [12–15]. Strong breed variation also exists in behavioral traits including herding, guarding and hunting [16], as well as personality traits (e.g. hypersocial behavior) [17] including boldness [18] and aggression [19]. The adoption of the ‘breed barrier rule’, i.e. that no dog may become a registered member of a breed unless both its dam and sire are registered members, has led to the establishment of breeds with highly restricted gene pools [20–22]. As a result, there is strong phenotypic homogeneity within all breeds [23]. Most breeds were established within the last 200 years [23,24] and were derived from small numbers of founders [24,25]. Consequently, the extraordinary phenotypic variation across dog breeds is accessible through analysis of only a modest number of genetic markers [3,26–29].

To create dogs with specific phenotypes, breeders often cross closely related individuals, and this particularly took place during the early formative years of many breeds. One consequence of this strategy has been an increased incidence of breed-specific genetic disease. A growing community has taken advantage of these observations to identify genes for canine maladies that recapitulate human disorders, many of which lack suitable mouse models. Indeed, with few exceptions, dogs experience the same common disorders as humans including cancer, heart disease, neurological disorders and diabetes (reviewed in: [20,21,30]). The underlying disease pathology is often similar to humans, as is the response to treatment and final outcomes (e.g. [31]). One additional consequence of the restrictive breeding programs that produced many modern breeds is the observed excess of recessive diseases, many of which have the potential to significantly advance our understanding of human orphan disorders, a benefit of no other medical model [32].

**Figure 2.**
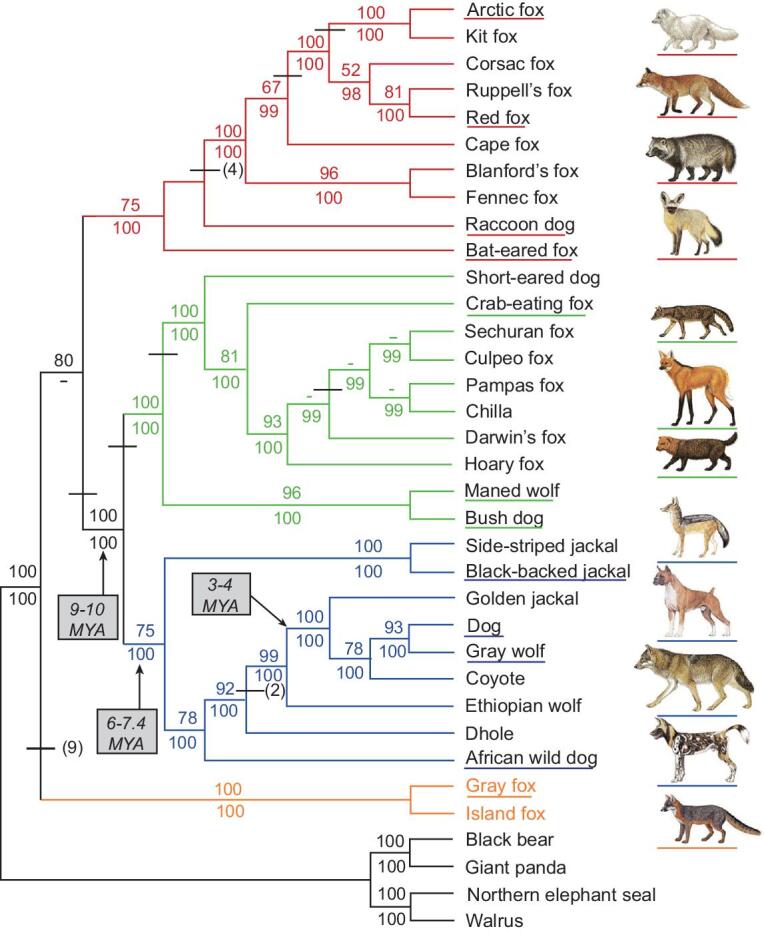
Phylogenetic tree shown is based on 15 kb of exon and intron sequence. Branch colors identify the red fox-like clade (red), South American clade (green), wolf-like clade (blue), and the gray and island fox clade (orange). Tree was constructed using maximum parsimony. Bootstrap values and Bayesian posterior probability values are listed above and below the internodes. Dashes indicate bootstrap values <50% or posterior probability values <95%. Species names are represented with the matching illustrations to the right. The figure is used with permission from Lindblad-Toh *et al.* [3].

One of the primary goals of this initiative is therefore to advance dogs as a model genetic species. Dogs were the first domesticated species and the only animal domesticated prior to the advent of agriculture [33]. In order to understand the range of genetic variability in dogs, it is crucial to investigate the entirety of canine evolution and domestication history (Fig. 2), which are tightly linked to that of humans [34–36]. As a result, strong selection on relatively few genes underlies many modern domestic phenotypes and may, in some cases, have led to genetic hitchhiking of deleterious alleles that contribute to disease risk [37]. In order to maximize the power of dogs as a genetic system for the study of human health and biology, and to comprehend the genetic basis for the myriad stages of domestication, it is crucial to ascertain the timing, geographical location and number of wolf populations that were involved during domestication [38,39].

## AIMS, SCOPE AND METHODS

### Aim 1: define the consequences of domestication on existing dog genomic diversity

#### Domestication

Numerous aspects of dog domestication are well accepted. There is a clear evidence, for example, that dogs were derived from gray wolves [40] and that no other canine species were involved during the initial phases of domestication [40–42]. In addition, the wolf population(s) that were involved in the early phases of domestication are likely extinct [42]. Despite the recent publication of numerous genetic studies of both modern and ancient dogs, there is as yet no firm consensus regarding either the timing [38,43,44] or location(s) [42,45–49] of domestication, the long-term effect of domestication on modern dog genomes [37,50], or even the number of independent wolf populations that were involved in the process [33,39]. The interpretation of the archaeological record has also been contentious. Though canid remains potentially derived from dogs have been excavated from Mesolithic contexts dated to 15 000 years before present in Europe and East Asia, the status of these remains as dogs or wolves is not easily resolved [33,51]. The characterization of genomic sequences from additional ancient and modern dog populations has the potential to resolve these controversies, and establish the early history of human–dog relationships.

Since their domestication, dogs have both adapted to novel environments as they dispersed across continents with their human companions [52–59], and been subjected to human selection for a diversity of occupations and aesthetic preferences. As a result, dogs are now globally dispersed, and they are the most abundant carnivore species in the world with a cosmopolitan distribution. Whole-genome sequencing (WGS) studies of numerous populations, breeds and wild canids are therefore crucial to advance our basic knowledge regarding the genetic outcomes of canine evolution (Fig. 2). Generating genome sequences on this scale will enable us to identify and characterize signals of selection related to domestication and dog breed formation at an unprecedented level of detail. Such studies will include not only the analysis of single nucleotide variants (SNVs), insertion deletions (indels) and copy number variants (CNVs), but also large structural variants, which are known for their key roles in aiding our understanding of the evolution of humans [28,60].

#### Admixture

Following domestication, dogs have traveled alongside humans across continents, often hybridizing with local wild canids. As a result, patterns of recent and ancient admixture among wild and domestic canid species are complex [38,42,60–63].

Dog10K will take advantage of this introgression history to explore the nature of selection, specifically in admixed populations. There are several examples of adaptive alleles in canids that have entered the population through admixture with other canid populations [54,57,62–65]. We will study adaptive introgression more generally and search for genomic regions enriched for ancestry from other populations. Furthermore, we will search for regions depleted of admixed ancestry. Such loci are less tolerant to admixture, and may contain genes that are important for speciation and domestication [38].

#### Selection

Using Dog10K data, we will expand maps of positive selection across the dog genome. More specifically, we will identify genes that differentiate modern dog and wolf populations [66]. Through integration of the sequence data derived from radiocarbon-dated individuals, we will use modeling techniques (e.g. [67]) to assess the strength and timing of selection over at least the last 15 000 years. Additionally, we will identify positive selection on genes within dog breeds through comparisons of haplotypes and linked variation among different breeds. The Dog10K data will also serve to tie signatures of selection with specific traits, local adaptation processes in wild canid populations, and provide a format for investigating the potential role of polygenic selection in canines, all of which are understudied.

The Dog10K effort to provide deep and comprehensive genome sequence data will further enable an improved inference of evolutionary rates in canids, a parameter that has been challenging to estimate [38,42,43]. This effort will reduce the confidence intervals surrounding inferred divergence times, effective population sizes and other demographic parameters. We will estimate mutation rates for a diverse array of molecular features, including SNVs, CNVs and mobile element insertions, and test whether mutational events cluster along the genome. These efforts will improve our understanding of the mutational process in dogs and wolves, and how mutations are affected by sequence composition, tandem repeats, CpG sites, chromatin accessibility and the unique nature of canine recombination hotspots [68]. To make these inferences, we will leverage patterns of linkage disequilibrium (LD) along with existing pedigree-based genetic maps. Lastly, the Dog10K data will provide a comprehensive catalog of rates of gene gain/loss in distinct canid populations and dog breeds relative to the canid phylogeny.

### Aim 2: dissect modern dog breed structure and morphological diversity

#### Modern breed structure

Having been subjected to centuries of strong human-mediated selection, dogs have evolved an extraordinary level of morphological and behavioral diversity (Fig. 1). By studying breeds, we can decipher the genetic basis of phenotype diversity and the consequences for numerous diseases. Our research will expand upon previous studies that have defined genes and/or specific variants for body size, skull shape, leg length, fur texture and pattern, and other traits (reviewed in [30]). An understanding of modern breed structure is critical when studying any locus in dogs, particularly those associated with disease, as closely related breeds are likely to share common susceptibility alleles due to shared, extensive tracks of homozygosity [63]. The closed structure of breeds yields disease phenotypes where few susceptibility alleles are likely to be causative, including complex diseases like cancer (reviewed in: [20,22,69,70]).

**Figure 3.**
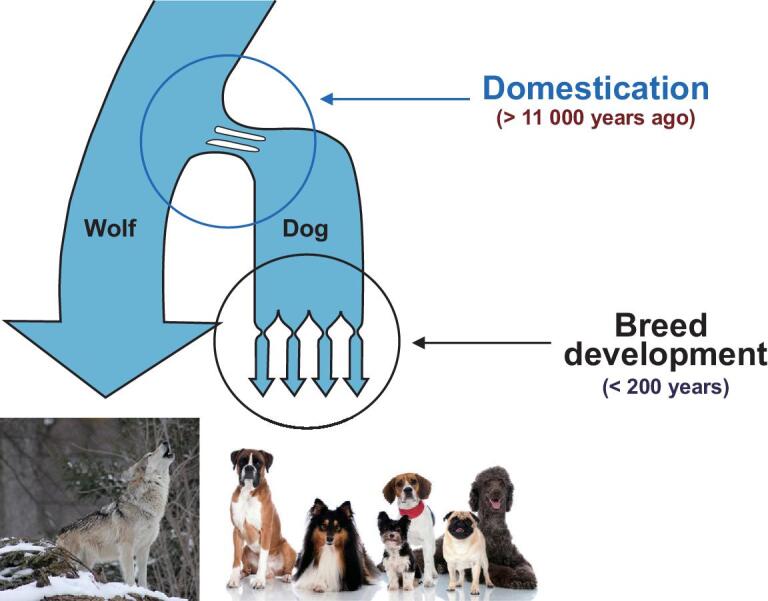
Multiple bottlenecks have shaped the structure of haplotypes and LD observed in modern breeds. Schematic indicates two bottlenecks that defined modern breeds. The first is believed to have occurred more than 11 000 years ago during domestication. The second encompasses many individual bottlenecks that occurred during primary breed formation about 200 years ago, producing the founders of the breeds observed today.

Each dog breed possesses a dense population structure and recognizable patterns of haplotype inheritance [3,63,71,72], which can both help and hinder attempts to map causal genetic variants in dogs. During breed development, where individual dogs were selectively bred to propagate desirable traits, the resultant population bottleneck led to large chromosomal regions that were concentrated within the new smaller populations (Fig. 3) [3,50,61]. This process has produced LD blocks that are 50-fold longer than those observed in human populations [3,73]. Initial measurements predicted that within-breed LD blocks could reach 2–5 megabases, but it is now established that LD blocks range from tens of kilobases across breeds to many megabases within breeds [74]. This substantial increase in LD block size permits the use of fewer genetic markers to capture patterns of selection and divergence than would be possible in species (such as humans) with smaller average LD blocks. The most recently developed canine array has approximately 650 000 single nucleotide polymorphisms (SNPs) (Affymetrix Infinium HD Ultra), which is a significant increase over the Illumina 170 000 SNP chip.

While canine genomics has evolved significantly with respect to family and association studies, the identification of functional variants remains difficult. Not unexpectedly, recessively acting alleles, gene–gene interactions and CNVs all contribute to complex phenotypes in dogs, and are not easily found through either segregation or association studies [75]. Single-breed studies often contain large blocks of LD, making the transition from locus to gene and, finally, to causative variant especially challenging (reviewed in [20–22]). In addition, statistical associations that include only a single breed may result in false-positive signals due to breed-specific population structure. The solution is to employ multiple breeds in a well-balanced association study, which can negate the impact of a single breed's demographic history and allow for greater cohort sizes [15,76]. Importantly, the use of SNPs or other markers derived from sequencing large numbers of diverse breeds can permit the identification of not just loci, but also genes and associative variants [4]. Greater numbers of individual dogs and breeds improve the resolution of association studies, since each additional breed reduces the inevitable skewing of results due to inherent phenotypic or phylogenetic imbalances between cases and controls.

**Figure 4.**
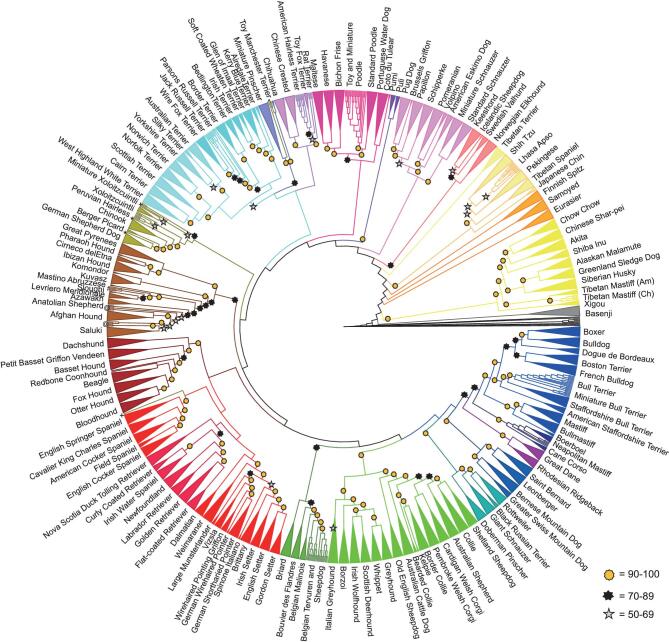
Neighbor-joining tree of 161 dog breeds. Cladogram showing relationships among 161 dog breeds that divide into 23 clades. Breeds that form unique clades are supported by 100% bootstraps and are combined into triangles. For all other branches, bootstrap values are ≥90% (gold star), 70%–89% (black star) and 50%–69% (silver star). The figure is used with permission from Parker *et al.* [63].

To take the greatest advantage of the dog model, it is critical to determine the relationships between dog breeds and the traits they share. Early studies of breed structure and relatedness failed to explain the mechanisms through which distinct breeds have developed, such as geographical separation and immigration, the role of hybridization and the timeline of breed formation. Building on previous studies [61,77], a bootstrapped cladogram using a neighbor-joining tree algorithm that defined 23 supported multi-breed clades representing geographical and occupational groupings was recently developed (Fig. 4) [63]. Identical-by-decent haplotype sharing was calculated using 1359 dogs from 161 breeds to assess introgression during breed development [63]. Individual instances of haplotype sharing between breeds with diverse phylogenetic backgrounds suggest that inter-clade crosses were carried out intentionally and often for specific reasons, such as the introduction of a new trait. By establishing a linear relationship between the total length of haplotype sharing and the age of known introgression events, undocumented crosses or divisions from older breeds that occurred within the last 200 years can be accurately estimated. These studies provide guidance regarding foundational breeds to select for the Dog10K sequencing efforts, particularly as it pertains to disease gene mapping.

**Figure 5.**
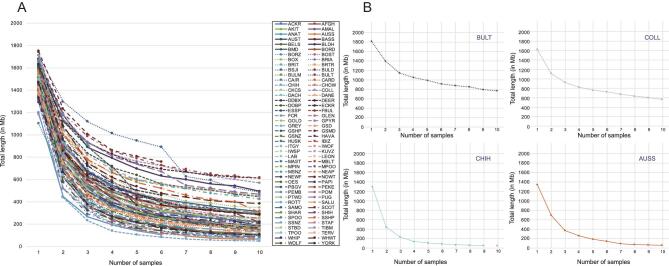
Shared regions of homozygosity (RoH) and length of homozygosity (LnH) data derived from SNP chip analyses. (A) represents each of 80 individual dog breeds, and displays the overall pattern of loss of private homozygosity beginning with one dog and expanding to 10 unrelated individuals. (B) illustrate homozygosity decay curves for a small subset of breeds at high levels of RoH and a low rate of decay [Bull Terrier (BULT) and Collie (COLL)], and a low level of RoH and a high rate of decay [Chihuahua (CHIH) and Australian Shepherd (AUSS)]. The figure is used with permission from Dreger *et al.* [71].

Dog10K design recommendations also consider the population structure for each breed that will be sequenced. Dreger *et al*. [71] used a panel of 170 000 SNPs to genotype 80 breeds in order to define single-breed patterns of homozygosity, shared homozygosity over 10 same-breed dogs and the rate at which any one dog will reduce the calculated shared homozygosity for its breed (Fig. 5). These efforts revealed that the breed-specific rate of homozygosity decay ranges over 3-fold, which can be used to estimate the number of dogs required to theoretically represent the entire amount of genetic variation within that breed. For example, a breed with a homozygosity decay value of 0.2 would require 18 dogs to reflect 99% of the within-breed variation. In general, the use of three unrelated dogs captures ∼85% of the variation in a registered breed [71]. These results provide guidance on the number of dogs of a given breed undergoing sequencing through Dog10K. We will also collect metadata on each dog whenever possible, including birth dates, registration numbers, standard morphological measures, disease histories, pedigree data and other data (Table 1).

**Figure 6.**
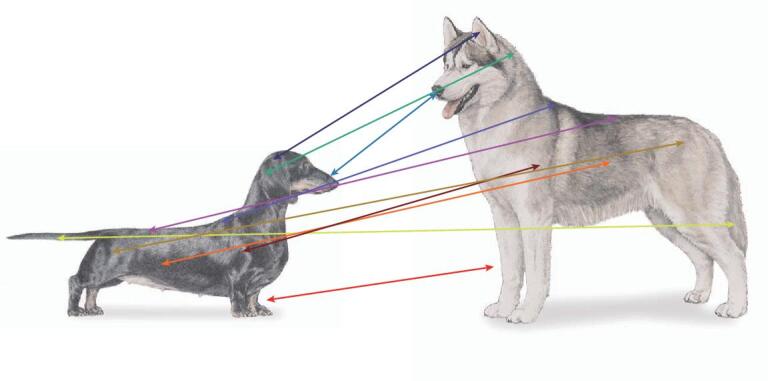
Multiple measures define dog breeds. In mapping traits such as body size, height or mass, measures of legs, skull, back, etc. need to be considered. The most accurate results will be derived from the most precise and greatest number of appropriate well-measured features. Because recognized breeds have well-established standards that encompass the above, breed standard data can often substitute for individual measures.

**Table 1.** Core metadata.

**Phase 1**	
Registered breeds (300 breeds, five per breed)	1500
Geographic distribution breeds (10 breeds, 50 per breed)	500
Most popular breeds (25 breeds, 50 per breed)	1250
Niche populations (20 populations, five per population)	100
Mixed breeds (30 populations, 10 per population)	300
Village dogs (100 populations, five per population)	500
**Phase 2**	
Ancient breeds (10 breeds, 100 dogs per breed)	1000
**Phase 3**	
Dog pedigrees (15 pedigrees, six per pedigree)	90
Wolf pedigrees (six lineages, six per lineage)	36
**Phase 4**	
Non-pedigreed wolves (20 populations, five per population)	100
Coyotes (three populations, five per population)	15
Golden jackals (three populations, five per population)	15
Wild Canids (six populations, five canids per population)	30
*De novo* assemblies (14 individuals)	14
**Total**	**5450**

**Table 2.** Initial sampling for Dog10K sequencing.

Documentation of approval by an ethics committee (if applicable)
Copy of pedigree certificate for registered individuals
Date of birth
Date of sample collection
Sex
Country of birth
Photo(s) to document phenotype
Laboratory or institute of sample origin
Sample type (e.g. blood, tissue type, hair) and storage buffer (e.g. ethylenediaminetetraacetic acid)
Optional: disease phenotypes; standardized X-rays; morphometrics; weight at time of sample collections

#### Morphological phenotypes

The first genome-wide SNP array-based association studies of morphology were published in 2010, linking over 60 traits to large genomic regions [26,27]. These results have been expanded several times [28,29], most recently using over 20 million SNPs and small indels culled from WGS of 722 canids [4]. Fine-scale studies have also identified specific

genes and/or variants associated with many morphological features, often by relying on strict breed standard measures, which have been shown to accurately reflect breed variance [18,78]. Individual measures are therefore not necessary to map breed-associated morphological traits.

Among the most-studied traits in dog breeds is body size, which is a composite of many features (Fig. 6). Large- and small-sized dog breeds differ in weight and some measures of size by nearly 40× (e.g. Great Dane and Chihuahua), a claim no other land mammal can make. A number of genes have been found to be major contributors to breed standard body weight (BSW), including *GHR, HMGA2, SMAD2, IGF1, IGF1R* and *STC2* [79–81], which account for 46%–52% of overall variance across modest and small breeds <41 kg (90 pounds). Variants in three genes on the X chromosome contribute to breeds with a BSW >41 kg (90 pounds) [5]. Among these are *IRS4* and *IGSF1*, both of which are involved in the thyroid hormone pathway and are associated with *IGF1R* signaling, obesity and body mass index in humans [82–84]. Also important in large body mass are variants in the *ACSL4* gene, which is associated with insulin resistance in humans. A similar role in dogs likely explains why the derived *ACSL4* variant is homozygous only in large ‘bulky’ dogs (e.g. English Mastiff and St. Bernard), while the ancestral allele is homozygous in large lean breeds (e.g. Irish Wolfhound and Greyhound). Overall, just 14 genes account for >90% of BSW in purebred dogs, with *IGF1* and *LCORL* being the largest contributors [4]. These studies highlight a recurring theme in dog genetics whereby a small number of genes of large effect control complex phenotypes, as opposed to many genes of small effect exerting similar levels of control, which is typical for human traits (e.g. >180 human body size loci [85,86]).

While latter studies of BSW in dogs were done using WGS data, initial studies were conducted using SNP arrays containing about 170 000 markers. Array-based studies often have the limitation of querying SNPs at low density across the genome with loci unrepresented by SNPs on the array. Furthermore, only a small fraction of the total SNP count on arrays is informative for every mapping study. Some investigators still rely on candidate gene analysis for such studies. One successful example is that of a 14 bp deletion in the *proopiomelanocortin* (*POMC*) gene, which has been shown to be important in obesity phenotypes in Labradors and Flat Coated Retrievers [87–89]. At least three other genes are associated with obesity in dogs [90], but it remains unknown how these influence BSW measures. These studies are valuable since they demonstrate that at least some of these genes (e.g. *G protein coupled receptor 120*) are associated with obesity in humans, and they show an ongoing role for candidate gene studies in dogs that can draw links to human health.

### Aim 3: explore the recurring theme of shared diseases between dogs and humans

Multiple studies have established the dog as a viable model for studies of disease susceptibility, progression, treatment response and outcomes (reviewed in [20,22,30,31,91]). Dog10K will expand that applicability by developing an exhaustive catalog of genetic variation to enable the generation of an accurate imputation panel for dog genome-wide association studies (GWAS), in much the same way that the 1000 Genomes Project has improved the study of low-frequency variants in human association studies. In addition, recent SNP array studies have indicated that significant allele frequency differences exist between sample collections of the same breeds from distinct geographical locations, calling for sample sequencing from different geographical regions to create the most accurate catalog possible.

Dog10K’s emphasis on providing WGS from aged, healthy individuals will also benefit disease studies. These data are often missing from current studies as a result of cost constraints, which have led investigators to preferentially sequence diseased individuals and rely on publicly available sequence databases to determine breed allele frequencies. However, the populations sampled in public databases are not representative of all breeds and data are lacking even for breeds where disease frequency is high. Dog10K will address this information gap by sequencing aged healthy individuals representing breeds whose relative risk for a heritable disease is high, such as a particular type of cancer [92–94], e.g. Bernese Mountain Dogs and histiocytic sarcoma [95–97], Scottish Terriers and bladder cancer [98], Golden Retrievers and lymphoma [99], and Irish Wolfhounds and osteosarcoma [100]. Equally important are breeds with a predisposition to cardiac issues, neurological, neuromuscular and autoimmune disorders, deafness and ocular disease, etc., and breeds that experience multiple distinct diseases at a high frequency. Reference sequence data on all breeds will provide the scientific community with an ability to perform matched WGS-based case-control analysis, either directly or through imputation. This aim is important, as it is the link between canine and human health that has generated the most interest on the part of the biomedical community.

WGS data can also be used to determine the genes that contain the lowest levels of polymorphism across canids. Such genes are likely to be under strong purifying selection in canids. We can intersect the resultant list with a similar database derived from humans. These comparisons will reveal the extent to which purifying selection has shifted across the mammalian phylogeny. The same dataset will be used to screen mutations assigned ‘variant of unknown significance’ status in human disease association studies. Variants found in the Dog10K catalog in breeds that are not at increased risk for the disease in question, or that are found frequently across breeds, are unlikely to be disease-associated, whereas those that are present in at-risk breeds not only provide useful data for human genetic studies, but also suggest an animal model for the development of therapeutics. The challenge will be in setting thresholds, as both neutral and disease alleles of varying types are nearing fixation in a variety of breeds. Despite that difficulty, Dog10K may benefit the human medical community by providing foundational data and resources for diseases of interest, for both human and veterinary disorders.

#### Behavior

Behavioral traits have come under recent scrutiny in dogs. It has become a recurring theme in dog genetics that studying dog breed phenotypes can reveal genes that, when heavily altered, cause human anomalous behaviors [101]. For instance, vonHoldt and colleagues recently showed that structural variants in genes associated with human Williams–Beuren syndrome, particularly *GTF2I* and *GTF2IRD1*, may contribute to behavioral differences between dogs and wolves [17]. In humans, a hemizygous deletion of this region causes delayed development, cognitive impairment, behavioral abnormalities and, most importantly, hypersociability. Increased levels of hypersociability may have been important during the domestication process that led to the emergence of dogs as companion animals. Indeed, a key phase in domestication appears to have been changes in social behavior and the corresponding genes (e.g. oxytocin receptor genes [102] and neural-related genes [103–105]).

The mapping of anomalous behavior will be a further goal of Dog10K and may be one situation where ‘affected’ dogs can be included without compromising other goals, as the resulting sequence is useful for mapping not only behavioral anomalies but also all other studies of demography, Mendelian disease and morphology. Obvious breeds for inclusion are those with obsessive compulsive disorders (OCD) [106] including the Bull Terrier, which is well known for its compulsive tail chasing [107,108], and Doberman Pinschers with their blanket- and flank-sucking behavior [109]. Genetic analyses suggest that the *CDH2, CTNNA2, ATXN1*, and *PGCP* genes are involved in OCD [110] and have led to the detection of four genes in related pathways in humans [111]. The first three genes mentioned above are themselves excellent candidates for the identification of additional human OCD genes, since they play a role in both brain development and synaptic plasticity. Further exploration of canine OCD by Dog10K may prove crucial for future studies that address human and animal mental health.

Since Dog10K will focus on mapping breed-specific behaviors, we face challenges in accurate phenotypic dissection of complex behavioral traits, especially those that are breed-related [112]. Incorporating experts in behavioral assessment and data storage will be key in disentangling the genetics of breed behaviors, and understanding how identical genes and pathways function in humans. Dog10K will therefore employ a behavioral scientist to collect and quantitate data. Finally, we are mindful that epigenetic variation also plays a highly relevant and underappreciated role in behavior, and focused studies will be needed to tackle related questions [113,114].

### Sampling scheme

The Dog10K sampling scheme encompasses multiple goals and numerous populations, ranging from the most strictly controlled breeds to lineages with loose associations to human settlements [51]. At the most intensive end of the spectrum of artificial selection are *registered breed dogs.* These have been developed through closed breeding lines and strong selection for appearance and function, a process that began during the Victorian era [115], have well-documented long-term pedigrees and clearly defined aesthetic breed standards by which individuals are judged [23]. Registered breeds capture most canine morphological variation and are key for identifying genotype–phenotype relationships.

A second sampling focus is on *niche* or *non-breed populations* of dogs that exist in communities throughout the world, each with a unique history that often mimics the settlement of humans in the region. These are in contrast to traditional or purebred breed dogs, and have been primarily and selectively bred to have specific occupations (e.g. herding, pointing, guarding and retrieving) that often constrain morphological or physical traits (e.g. long legs, particular coat color and muzzle shape). Many such breeds are often supposed to possess origins in ‘antiquity’ and were foundational for developing the modern breeds, although it is well established that many so-called ‘ancient’ breeds are in fact modern constructions with established histories [51,63].

The majority of the global population of dogs are not selectively bred, but nevertheless live in some degree of association with humans (e.g. guarding or companion versus scavengers on the margin of human society) [49]. Referred to as *free-breeding dogs*, the reproductive success and survival of these indigenous regional populations of dogs is not strictly determined by humans. We distinguished between two major types of free-breeding dogs: *village dogs* that live in rural regions and are often, but not always, relatively unaffected by admixture from other regions, and free-breeding dogs that inhabit major metropolitan areas termed *street dogs* [116]. Further, we define an additional category of *feral dogs*, which are those that are largely under natural selection and have little human interaction. While the ancestors of feral dogs were domestic, these populations exist today largely as wild animals (e.g. Australian Dingoes).

Our studies on the genomic impact of canine domestication will rely on extensive sampling across *wild canid lineages* (Fig. 2). There is substantial phenotypic variation across wolf populations driven by local adaptation and, in some cases, introgression with dogs (e.g. coat color) [57,64]. The inclusion of diverse wild canids, particularly gray wolves, is crucial for understanding both their demographic history and trait evolution [42,103]. Dog10K will utilize a phylogenetic outgroup species (e.g. cat [117]) to improve the detection of accelerated evolution within branches of *Canis* and identify derived alleles, a crucial consideration for many methods of inference in evolutionary genomics. An appropriate evolutionary outgroup would share little, if any, segregating variation with dogs.

### Sequencing effort

Sequencing will be carried out in four phases. The selection of the first 5450 canids to be sequenced is described below (Table 2). Overall, we will generate moderate-coverage genomes (≥20-fold average) to curate the breadth of contemporary genomic variation in dogs and to investigate how this variation leads to phenotypic variation.

#### Phase 1

Phase 1 will include the sampling of at least five unrelated dogs across 300 distinct registered breeds that represent the spectrum of modern breed dog populations worldwide (*n*_breeds_^1^ = 1500). Inclusion of a specific breed will be determined by the demographic relationship to other breeds, the hypothesized strength of artificial selection for derived phenotypes (example: body size in Great Danes, brachycephaly in Pugs and olfaction in Bloodhounds) and disease susceptibility. In cases where individual breeds possess strong population structure, e.g. divergence between populations in US and in other continents, as in the Shar-Pei, we will include at least 10 individuals in order to capture the genetic diversity in each subpopulation. We predict this to be the case for an additional 50 breeds (*n*_breeds_^2^ = 500). In addition, we will select 25 popular breeds (e.g. German Shepherd Dog, Golden Retriever, Bulldog and Poodle) for which we will thoroughly explore intra-breed variation by sequencing a minimum of 50 dogs in each (*n*_breeds_^3^ = 1250). We will emphasize individual selection to maximize phenotypic variation (i.e. disease susceptibility and morphology) and minimize relatedness. This has already been done for the Yorkshire Terrier, and is available in the public 722 WGS data described below.

Though most of canine phenotypic variation is found within the registered breed dogs, many specialized traits are uniquely found among isolated indigenous populations. Thus, we will additionally sample five unrelated individuals from 20 niche or non-breed populations (*n*_niche_ = 100). Further, since 47% of all dogs in the US are categorized as either mixed-breed or designer-breed dogs [118], we will sequence 10 unrelated ‘mixed’ breed individuals from 30 different locations (*n*_mixed_ = 300). Free-breeding indigenous, village and street dogs possess more genetic variability than both breed and ‘mixed’ dogs, so we will include five unrelated individuals from 100 such dog populations across six continents, with an emphasis on indigenous village dog populations showing geographical signatures consistent with historical diversity instead of modern admixture (*n*_village_ = 500). We have already sequenced a total of 298 village dogs and the total for phase 1 will include 4150 samples.

#### Phase 2

Phase 2 (Table 2) will generate data from an additional 1000 samples, focusing on non-breed dogs from geographical regions that have not been systematically sampled. These include: East/Central Asia, the High Arctic, the Middle East, Eastern Europe, Africa and Island Southeast Asia. We will generate ≥20× genomes of 100 unrelated individuals of 10 specified breeds, which are yet to be determined (total *n*_ancient_ = 1000), and this number will likely increase.

#### Phase 3

In phase 3, we will sequence extended pedigrees of canids (including at least three generations) to ≥50× per individual. We estimate that this will encompass six dogs each for 15 pedigrees (*n*_pedigree_dog_ = 90). In addition to dogs, we will sequence gray wolves from Yellowstone with genetically verified pedigrees [119], including six wolves per founder lineage, with approximately six lineages available (*n*_pedigree_wolf_ = 36) for an initial total of 126 wolves. These are distinct from those mentioned above. This study design will reduce false-positive mutational events due to sequencing errors. Further, it will limit the false-negative rate by ensuring that we can reliably call heterozygous genotypes. Because pedigree data are so extensive on these individuals, the data will also allow the estimation of fine-scale recombination and mutation rates across the genome in different canid populations.

#### Phase 4

In phase 4, we will capture the genomic variation among wild relatives of dogs and their evolutionary relationships. We will sequence ≥20× genomes of five unrelated gray wolves from 20 populations (*n*_wolf_ = 100), five unrelated coyotes and golden jackals from three populations each (*n*_coyote_ = 15 and *n*_golden_jackal_ = 15), and five unrelated wild canids including each of the following species: golden wolves, Ethiopian wolf, dhole, black-backed jackal, African wild dog and side-striped jackal (*n*_wild_canid_ = 30). All will be sequenced to 20× and we expect this phase to expand significantly as the project progresses to include, for instance, gray and red foxes.

Prior to sample collection, all dog owners must sign standard Animal Care and Use Consent forms providing signed permission for the collection of a blood sample, pedigree data and registration number (if available), demographic history (if relevant), owner contact information, consent to re-contact owner, medical history and pedigree data for privately owned dogs as listed (Table 1). While blood samples are desirable, samples from some dogs may only be available using buccal swabs.

The numbers presented here for phases 1 and 2 are in addition to the 722 WGS samples already released and cataloged by members of the community, producing over 91 million variants (https://research.nhgri.nih.gov/dog_genome/data_release/index.shtml [4]; see also ncbi.nlm.nih.gov/sra). All of the sequence data produced and made public by the general canine genome community will be integrated into the total dataset. The initial 5450 samples selected here (Table 2) represent about one-half of the proposed 10 000 samples. Subsequent expansion will focus on rare breeds, comprehensive village dog sampling, disease phenotypes, robust within-breed sampling and geographical representation to highlight subtle phenotypic variation. Members of the scientific or lay community can nominate breeds based on these criteria.

Dog10K also plans to establish high-quality reference genomes that will be *de novo*-assembled using a variety of technologies including Pacific Biosciences long-reads (100×), Bacterial Artificial Chromosome end sequencing, optical-mapping (Bionano Saphyr), phased haplotypes (10X genomics 60×) and chromosome conformation (Hi-C), etc. We will begin with a *de novo* assembly of the original Boxer from which the 2005 sequencing study was carried out [3]. The production of this assembly, combined with a realignment of currently available genomes, will improve the reference dog genome by utilizing gap-filling and sequence error correction to facilitate the identification of critical regions constituting genic and regulatory elements. Furthermore, we will generate a single *de novo*-assembled genome for each of the following: outgroup species, gray wolf, coyote, golden wolf, African village dog, South Chinese village dog, Siberian or Alaskan dog, Middle East, Indian village and a Golden Retriever. We will begin with this initial set, but expect that more will be added. Ongoing *de novo* assembly efforts in the community also include the Labrador Retriever, German Shepherd Dog, Great Dane, Rottweiler, Collie and others. For all phases, the final sample numbers will fluctuate depending on sample availability and the advancing priorities of the community.

### Online resources and data sharing

Raw sequence data along with sample metadata, including sample name, breed, sampling location and any disease phenotypes, will be deposited in the Genome Sequence Archive (GSA) (http://gsa.big.ac.cn/), as well the International Nucleotide Sequence Database Collaboration (INSDC, a collaboration that includes DNA Data Bank of Japan (DDBJ), European Molecular Biology Laboratory's European Bioinformatics Institute (EMBL-EBI) and National Center for Biotechnology Information (NCBI)), which will enable automatic synchronization across the three repositories. Variant call files, as well information on coverage and genome quality control, will be made available through the project website and individually submitted to the Ensembl Variation database. Revised genome assemblies and associated annotations will be deposited in the GSA and INSDC databases, along with required files for exploring the data through existing genome browsers. The web sites that will be maintained as part of this project include: the Dog10K website (http://www.dog10kgenomes.org), the iDog database [120] (http://bigd.big.ac.cn/idog), the DogSD database [121] (http://dogsd.big.ac.cn/), https://research.nhgri.nih.gov/dog_genome/data_release/index.shtml, https://ncbi.nlm.nih.gov/sra, https://midgard.nhgri.nih.gov/dog_genome/index.shtml and the European Consortium; NCBI and European Nucleotide Archive (ENA) for FASTQ files.

## CONCLUSION

The development of the Dog10K resource will significantly advance studies of human-relevant disease in domestic dogs. Not only will dense sequencing of key breeds enhance the ability to find susceptibility loci, genes and variants, but the ensuing results are more likely to mimic those of humans than those observed in more common model systems such as mice, where disease states are often induced rather than naturally occurring. The ultimate legacy of the dog as a biomedical model for human disease lies in its translational potential.

The evolutionary history of dogs is controversial and requires complete genomes to provide sufficient resolving power to test hypotheses related to demographic history, admixture and selection during dog domestication. Furthermore, a population genomics approach to understanding the constraints on variation and the effects of deleterious mutations is in its infancy, and will benefit from wider population sampling. Full genomes from diverse populations will provide new data to test ideas about the distribution of the effects of selection, which are integral to evolutionary models of demography and adaptation. Finally, the landscape of recombination is a critical issue in canines who lack a functional version of the nearly universal protein PRDM9, which is associated with recombination hotspots. Therefore, the study of canine genomes will allow unique insights into the alternative mechanisms by which recombination is maintained.

## Alphabetical listing of Dog10K Consortium members (* indicates primary organizers)

Catherine André: University of Rennes, CNRS, IGDR, UMR6290 - F-3500 Rennes, France, christophe.hitte@univ-rennes1.fr
Erik Axelsson: Department of Medical Biochemistry and Microbiology, Uppsala University, Uppsala biomedicinska centrum BMC, Husarg. 3 75237 Uppsala, Sweden, erik.axelsson@imbim.uu.se
Adam Boyko: Department of Biomedical Sciences, Cornell University College of Veterinary Medicine, Veterinary Research Tower T8016A, Box 17, Ithaca, NY 14853, USA, boyko@cornell.edu
Brian W. Davis: College of Veterinary Medicine and Biomedical Sciences, Texas A&M University, College Station, TX, USA, bdavis@cvm.tamu.edu
Oliver Forman: Genetics Research Unit, Mars Inc., Cambridge, UK.
Laurent Frantz: School of Biological and Chemical Sciences, Queen Mary University of London, London, UK, Laurent.frantz@qmul.ac.uk
Christophe Hitte: University of Rennes, CNRS, IGDR - UMR6290, F-3500 Rennes, France, hitte@univ-rennes1.fr
Vidhya Jagannathan: Institute of Genetics, Vetsuisse Faculty, University of Bern, Bern, Switzerland, vidhya.jagannathan.@vetsuisse.unibe.ch
Elinor Karlsson: Bioinformatics and Integrative Biology at University of Massachusetts Medical School, and Vertebrate Genomics and Genetics Group at the Broad Institute of the Massachusetts Institute of Technology and Harvard, Boston, MA, USA, elinor.broadinstitute.org.
Jeffrey Kidd: Departments of Human Genetics and Computational Medicine and Bioinformatics, University of Michigan Medical School, 3726A Med Sci II, Ann Arbor, MI 48109, USA, jmkidd@umich.edu
Greger Larson: Research Laboratory for Archaeology and the History of Art, Dysons Perrins Building, South Parks Road, University of Oxford, Oxford, OX1 3QY, UK, greger.larson@arch.ox.ac.uk
Tosso Leeb: Institute of Genetics, Vetsuisse Faculty, University of Bern, Bern, Switzerland, tosso.leeb@vetsuisse.unibe.ch
Kerstin Lindblad-Toh: Science for Life Laboratory, Department of Medical Biochemistry and Microbiology, Uppsala University, Uppsala, Sweden and Broad Institute of the Massachusetts Institute of Technology and Harvard, Cambridge, MA 02142, USA, kersli@broadinstitute.org.
Hannes Lohi: Research Programs Unit, Molecular Neurology and Department of Veterinary Biosciences, University of Helsinki and Folkhälsan Institute of Genetics, PO Box 63, Helsinki, Finland.
Kirk E. Lohmueller: Ecology and Evolutionary Biology, University of California, Los Angeles, Los Angeles, CA 90095, USA klohmueller@ucla.edu
Tomas Marques-Bonet: Institut Biologia Evolutiva (Universitat Pompeu Fabra/CSIC) ICREA, Barcelona, Spain, tomas.marques.@upf.edu
Catherine Mellersh: Animal Health Trust, Lanwades Park, Kentford, Newmarket, Suffolk, CB8 7UU, UK, cathryn.mellersh@aht.org.uk
*Elaine A. Ostrander: Room 5351, Building 50, National Human Genome Research Institute, National Institutes of Health, 50 South Drive, Bethesda, MD 20892, USA, eostrand@mail.nih.gov
Peter Savolainen: School of Biotechnology, Science for Life Laboratory, Royal Institute of Technology in Stockholm, PO Box 1031, SE-171 21, Solna, Sweden, savo@biotech.kth.se
Robert Schnabel: Division of Animal Sciences, Informatics Institute, University of Missouri-Columbia, Columbia, MO 65211, USA
Bridgett M. vonHoldt: Ecology and Evolutionary Biology, Princeton University, Princeton, NJ 08544, USA, vonHoldt@princeton.edu
*Guo-Dong Wang: State Key Laboratory of Genetic Resources and Evolution, Kunming Institute of Zoology, Chinese Academy of Sciences, Kunming 650223, China, wanggd@mail.kiz.ac.cn
*Robert K. Wayne: Ecology and Evolutionary Biology, University of California, Los Angeles, Los Angeles, CA 90095, USA, rwayne@ucla.edu
Ziheng Yang, Department of Genetics, University College London, London, WC1E 6BT, UK, z.yang@ucl.ad.uk
Weiwei Zhai: Key Laboratory of Zoological Systematics and Evolution, Institute of Zoology, Chinese Academy of Sciences, Beijing 100101, P.R. China, wwzhai@ntu.edu.sg
*Ya-Ping Zhang: State Key Laboratory of Genetic Resources and Evolution, Kunming Institute of Zoology, Chinese Academy of Sciences, Kunming 650223, China, zhangyp@mail.kiz.ac.cn
